# Sociocultural and Behavioral Features of Anticipated COVID-19 Vaccine Acceptance in Papua New Guinea: Protocol for a Mixed Methods Study

**DOI:** 10.2196/44664

**Published:** 2023-05-02

**Authors:** Joseph Gnanouday Giduthuri, Clement Manineng, Elisabeth Schuele

**Affiliations:** 1 Population Health and Demography Research Unit Papua New Guinea Institute of Medical Research Goroka Papua New Guinea; 2 Swiss Tropical and Public Health Institute University of Basel Basel Switzerland; 3 Faculty of Medical and Health Sciences Divine Word University Madang Papua New Guinea

**Keywords:** COVID-19 vaccine, vaccine acceptance, vaccine demand, community views on vaccine, vaccine stakeholders, health care workers, Papua New Guinea, emergency preparedness, public health intervention, COVID-19, health promotion, community health, Melanesian culture, vaccine uptake, vaccine awareness, vaccine priorities

## Abstract

**Background:**

COVID-19 was characterized by the World Health Organization (WHO) as a pandemic in 2020. Papua New Guinea (PNG) has remained on high alert ever since, and its National Control Centre continues to coordinate national preparedness and response measures, guided by its Emergency Preparedness and Response Plan for COVID-19. As part of the WHO and the Global Alliance for Vaccines and Immunization's COVID-19 Vaccines Global Access (COVAX) program, PNG received several shipments of COVID-19 vaccine doses. A nationwide vaccine rollout for COVID-19 was initiated in PNG in May 2021. Despite the availability of vaccines and the capacity of health systems to vaccinate frontline workers and community members, including high-risk groups, there are still critical issues related to vaccine safety, confidence, and acceptance to ensure the effectiveness of the COVID-19 vaccination campaign. Evidence from studies on COVID-19 vaccine acceptance and demand in low- and middle-income countries (LMICs) suggests that sociocultural characteristics of the community and the behaviors of different vaccine stakeholders, including vaccine recipients, vaccine providers, and policymakers, determine the effectiveness of vaccination interventions or strategies.

**Objective:**

This study will examine sociocultural determinants of anticipated acceptance of the COVID-19 vaccine in urban and rural areas of different regions in PNG and health care providers’ views on vaccine acceptance.

**Methods:**

The study design uses a mixed methods approach in PNG’s coastal and highlands regions. The first research activity will use a qualitative methodology with an epistemological foundation based on constructivism. This design elicits and listens to community members’ accounts of ways culture is a rich resource that provides meaning to the COVID-19 pandemic; the design also measures adherence to *niupela pasin* (“new normal” in Tok Pidgin) and vaccination acceptance. The second activity will be a cross-sectional survey to assess the distribution of features of vaccine acceptance, priorities, and practices. The third activity will be in-depth interviews of health care providers actively involved in either COVID-19 clinical management or public health–related pandemic control activities.

**Results:**

The project proposal has been reviewed and approved by the Medical Research Advisory Committee of Papua New Guinea. Qualitative data collection started in December 2022, and the survey will begin in May 2023. The findings will be disseminated to the participating communities later this year, followed by publication.

**Conclusions:**

The proposed research on community views and experiences concerning sociocultural and behavioral features of acceptance of the vaccine will provide a better understanding of communication and education needs for vaccine action for COVID-19 control in PNG and other LMICs. The research also considers the influence of health care providers’ and policy makers’ roles in the awareness and use of the COVID-19 vaccine.

**International Registered Report Identifier (IRRID):**

PRR1-10.2196/44664

## Introduction

### Background

COVID-19 affected almost every country in the Pacific region, including Papua New Guinea (PNG). As of August 2022, a total of 45,133 confirmed COVID-19 cases were reported in PNG, resulting in 668 deaths [1]. However, these numbers may be an underestimate caused by nonoperational information systems in rural areas. Globally, as a pandemic preventive strategy, countries have been using nonpharmaceutical interventions (NPIs), such as enforced use of face masks, hand sanitization, physical distancing, travel restrictions, avoidance of large gatherings, school closures, and partial or complete lockdowns. [2]. The health department in PNG has introduced the *niupela pasin* (“new normal” in Tok Pidgin) policy, referring to the changes in behavior and habits that individuals and communities need to adopt to prevent the spread of the virus [3]. Despite these NPIs and policies, there have been increased new cases and deaths each day throughout the world.

### COVID-19 Vaccines

Vaccines are the most reliable and effective public health intervention to prevent many infectious diseases [4,5]. Other interventions, like developing practical diagnostic tools and therapeutic drugs, also help clinical management and reduce mortality and morbidity. Therefore, developing and producing safe and effective vaccines remains one of the essential tools for ending the COVID-19 pandemic, along with NPIs [6]. In collaboration with global research institutions, the World Health Organization (WHO) and other global agencies accelerated the development process of COVID-19 vaccines while maintaining the highest standards of safety [7]. The WHO emergency use listing approved several vaccines developed on different platforms. The COVID-19 Vaccines Global Access (COVAX) program is a groundbreaking collaboration between the WHO, the United Nations Children’s Fund (UNICEF), the Global Alliance for Vaccines and Immunization (GAVI), and the Coalition for Epidemic Preparedness Innovations (CEPI) to accelerate the development and production of vaccines and equitable access to them for every nation in the world [8]. So far, the COVAX program has provided 71 million vaccine doses to 125 participating countries [9]. Many countries have initiated vaccine rollouts at a national level since 2021. However, with equitable access to and availability of COVID-19 vaccines, the issue of vaccine acceptance and uptake among different high-risk groups, including frontline health workers and the general population, remains critical.

### Research on COVID-19 Vaccine Acceptance

Although vaccines are generally well-received, the fast-tracking of COVID-19 vaccine development procedures and the use of new platforms, such as RNA-based vaccines, has created some doubts about vaccine safety and reduced confidence. Vaccine confidence can be defined as the trust and willingness of individuals and communities to accept and use vaccines, and it is important to measure vaccine confidence to identify and address community concerns about vaccination [10]. In general, vaccine hesitancy undermines vaccine acceptance and demand. The WHO’s Strategic Advisory Group of Experts (SAGE) on immunization defined vaccine hesitancy as a delay in acceptance or refusal of vaccination despite the availability of vaccination services [11,12]. It is crucial to assess the determinants of vaccine acceptance among vaccine recipients to deal with vaccine hesitancy.

Almost all countries in the world prioritized frontline workers for COVID-19 vaccination. However, research suggests that health care workers’ anticipated COVID-19 vaccine acceptance rates vary from low to high [13-15]. A global survey based on 13,426 participants from 19 countries showed that global acceptance of COVID-19 vaccines ranges between 54.8% in Russia to 88.6% in China [16]. In a narrative review of 15 studies, frequently given reasons for refusing the COVID-19 vaccine were related to the fast-tracked vaccine development and vaccine safety and effectiveness [17]. Nevertheless, the WHO has stated that vaccine safety was not compromised by fast-tracking development [7]. With careful evidence-based communication strategies, it is possible to convey the safety of COVID-19 vaccines to the general population.

### Public Health Priority of COVID-19 Vaccines in PNG

PNG is the largest country in the Pacific region. The health system of PNG has been struggling for decades to provide universal access to quality services [18]. Nevertheless, PNG has remained on high alert ever since COVID-19 was characterized by the WHO as a pandemic in 2020, and its National Control Centre (NCC) continues to coordinate national preparedness and response measures guided by its Emergency Preparedness and Response Plan for COVID-19 [19]. As in many low- and middle-countries (LMICs), even PNG, vaccination is the only way of protecting frontline workers and community members during the COVID-19 pandemic. As part of the COVAX program, 588,000 COVID vaccine doses were allocated for PNG. The National Department of Health initiated a nationwide vaccine rollout for COVID-19 on May 4, 2021 [20]. So far, more than 348,938 doses have been administered in 22 provinces of PNG [1].

### Rationale for This Research

Despite the availability of vaccines and the capacity of health systems to vaccinate frontline health care workers and community members, issues related to vaccine safety, confidence, and acceptance remain critical for the effectiveness of the COVID-19 vaccination campaign. Evidence from studies on differing vaccine acceptance in LMICs suggests that sociocultural factors in the community and the behaviors of different vaccine stakeholders, including vaccine recipients, vaccine providers, and policy makers, determine the effectiveness of vaccination interventions or strategies. So far, no literature exists on determinants of COVID-19 vaccine acceptance in the PNG context.

Understanding the local context is critical for an effective vaccination program at the community level. The proposed research on community views and experiences concerning sociocultural and behavioral features of anticipated vaccine acceptance will enable a better understanding of communication and education needs for vaccine action for COVID-19 control in PNG and other LMICs. Our pilot study, which explored views on COVID-19 vaccines among students (n=90) with different cultural backgrounds at the Faculty of Medicine and Health Sciences of Divine Word University, indicated 50% tentative acceptance and highlighted the role of the media in determining vaccine acceptance.

### Objectives

This study will examine sociocultural determinants of anticipated acceptance of the COVID-19 vaccine in urban and rural areas of different regions in PNG. The following are the specific study objectives: (1) understand community members’ accounts of experiences, meaning, and behavior concerning the COVID-19 pandemic, as well as their awareness of and adherence to *niupela pasin* and their perceptions of vaccines based on Melanesian culture, and (2) assess the role of health care providers in determining COVID-19 vaccine acceptance in the coastal and highlands regions, considering their awareness, acceptance and personal use, and vaccine recommendation practices.

## Methods

### Study Design

The research project will use a mixed methods design. The first research component will use a qualitative methodology with an epistemological foundation that is based on constructivism and embraces meaningful reality as it is socially constructed [21]. This design elicits and listens to community members’ accounts of ways culture is a rich source of meaning in the COVID-19 pandemic, their adherence to *niupela pasin*, and their vaccination acceptance. In the context of health, adherence to the new normal is an indicator of the behavior of communities toward their health and is important for understanding these communities. This design will proceed with individual, in-depth, semistructured interviews with community members, community opinion leaders, and church leaders.

The second component, a cross-sectional survey, will use data collected from ethnographic key informant interviews to develop an instrument with open-ended and closed questions. The survey design adapts the WHO immunization and vaccine-related implementation research advisory committee’s vaccine stakeholder framework to assess the awareness, priorities, and practices of community members. This design includes an integrated quantitative and qualitative methods approach, and the data collected will indicate the distribution of key features of vaccine acceptance and demand [22].

The third component, semistructured interviews with health care providers actively involved in either COVID-19 clinical management or public health–related pandemic control activities, will determine vaccine acceptance factors. This component will focus on assessing health care providers’ views on personal vaccine acceptance and their role in facilitating community vaccination.

### Study Sites

This study will proceed in urban and rural areas in the coastal and highlands regions of PNG. Most of the PNG population lives in rural areas (85%), and the economy is primarily agricultural. We selected the Momase region as the coastal region in our study. The Momase region is composed of 4 constitutional provinces with a population over 1.8 million (2011 census). Madang is one of the provinces in this region; it has a population of 493,906 (2011 census). The study will be conducted in urban and rural villages in Madang district.

The Highlands region is the most densely populated region of PNG, with 23% of the total PNG population (2011 census). This region is composed of 7 provinces. The Eastern Highlands province is in the central highland’s cordillera and has a population of 579,825 (2011 census). Goroka is the capital of this province. The study will be conducted in Goroka and in rural villages of the Lufa district. These study sites are culturally and linguistically diverse. Tok Pidgin is the lingua franca.

### Study Groups and Sampling

#### Community Members

The criteria for including community members in interviews and the cultural epidemiological survey are being aged between 18 to 65 years and being cognitively able and willing to participate. The study will be conducted in either English or Tok Pidgin and participants should be able to communicate in either of these languages.

#### In-depth Interviews

A purposive sampling strategy will be used for recruiting participants for in-depth interviews. The sample will be distributed equally among male and female participants (ie, a 50% gender distribution). Community members are key stakeholders in adhering to *niupela pasin* and providing meaning to vaccine acceptance and demand. Starting with exploring and understanding their views is therefore critical.

A collaborating team of research assistants at each study site will initially meet local communities in urban and rural sites to establish rapport. Community opinion leaders and leaders of formal and informal community groups will be contacted, and we will explain the purpose and anticipated value of the study. Further, we will request their participation and assistance to facilitate community participation. Engaging these urban and rural opinion leaders and enlisting their cooperation will facilitate the planning and implementation of in-depth interviews. We will interview church leaders from the selected study sites.

The team of research assistants will go from house to house in parts of the selected study area to identify potential participants and achieve the desired sample compositions of urban and rural and male and female participants. The team will explain the purpose of the visit, solicit potential participants’ willingness to participate in the study, and, if they agree, obtain their informed consent (one-to-one) in confidence and with due attention to privacy. A total of 48 in-depth interviews will be conducted (12 community opinion leaders, 24 community members, and 12 church leaders). All interviews will be audio-recorded. The duration of each interview will be approximately 30 to 45 minutes.

### Vaccine Acceptance Survey

A random sampling strategy will be used to select the participants for this survey. We plan to use either the voters’ list or household listings to randomize the sample at an individual level or use village maps to randomize households to select the eligible participants. This will be achieved in collaboration with the provincial health authority (PHA). However, randomization depends on the availability of these resources. Otherwise, a convenience sample will be selected from the urban and rural areas of the selected study sites, stratified by gender (with a 50% distribution) and age group (18-40 years and 41-65 years). Trained research assistants working in 2 teams will conduct the survey, one interviewing and the other responsible for maintaining the data records. Approximately 360 interviews will be conducted (180 in each of the two regions. We calculated the sample size at a 5% margin of error with a 95% confidence level and a 50% response distribution.

### Health Care Providers

Health care providers involved in either COVID-19 clinical management or public health–related pandemic control activities in the selected study sites will be interviewed. We will identify key informant health care providers during earlier stages of the study based on respondent-provided information that identifies health care professionals engaged in relevant activities.

This sample will be selected purposively, and a question guide will guide the in-depth interview. The rationale for using in-depth interviews is to allow health care professionals to share their experiences and views privately without having another person in the room. A total of 16 interviews will be conducted with clinicians, health extension officers, nurses, midwives, and community health workers in health facilities (government and church-based), including provincial hospitals and health centers of urban and rural study areas.

### Data Management and Analysis Plan

The study instruments were pilot-tested during training workshops for validity and feasibility. The interview guides for community members and health care workers were developed after consulting experts in the field and the literature. They were pilot-tested with potential participants for rigor and credibility. For the quantitative survey, we adapted an instrument developed by one of the authors for a vaccine acceptance study in India. After completing the qualitative component, we will further develop this questionnaire to ensure feasibility in the study context. This instrument will be tested for content and face validity. All the interview guides and initial questionnaires were developed in English and translated into Tok Pidgin. All instruments were then back translated to English for accuracy.

### Data Collection

In-depth interviews will be audio-recorded, and notes will be taken by the field investigators and research assistants. Qualitative data from in-depth interviews will be transcribed verbatim, translated into English, and imported into MAXQDA (version 2020; VERBI Software) for analysis.

The data collection and management of cultural epidemiology will be done using the Open Data Kit (ODK) tool (GetODK). Data will be collected digitally on an Android tablet using the ODK Collect application. Later, the data will be uploaded to a cloud server. From there, the data set will be downloaded as an Excel (Microsoft Corp) spreadsheet.

### Data Security

All interviews will be recorded for documentation. The information recorded is confidential. Data sheets, audio records, and transcripts will have unique identifier codes. They will not bear the participant’s name. Soft copies (ie, audio records and transcripts) will be stored electronically on password-protected external hard disks or drives. Hard copies (ie, data sheets) will be stored under lock and key at the faculty office. Informed consent forms will bear participants’ names and signatures. Therefore, they will be kept under lock and key separately from data sheets, audio records, and transcripts. They will be accessible through only the principal investigator or coinvestigators, with lists linking unique identifier codes to names. Data sheets, audio records, transcripts, and informed consent forms will be destroyed (by incineration or digital erasure) 3 years after study completion.

### Analysis Plan

The primary goal of the study is to clarify descriptive, comparative, and analytic accounts of explanatory models of COVID-19 and how they explain awareness, acceptance, and use of vaccines. Comparative analysis will focus on questions of gender and rural-urban features of the COVID-19 pandemic and determinants of anticipated acceptance or refusal of COVID-19 vaccines.

### Qualitative Analysis

The in-depth interviews of community members will provide their views on the risk of contracting the virus, behavior to prevent the virus, experience and meaning of COVID-19, and their perception of COVID-19 vaccination. The interviews with health care workers will provide their views about COVID-19 vaccine priorities and practices and their recommendations for promoting the rollout of vaccination. All these in-depth interviews will be transcribed verbatim, verified against the audio tapes, and translated into English. The English transcripts will then be imported into MAXQDA software for managing the data. Thematic analysis will be used as an inductive and deductive process. Analysis involves familiarization with the data, generation of codes, and discovery of initial themes. Themes will be refined through an interactive process, and a coding framework will be developed that reflects the core themes [23]. A team of research assistants with author ES will develop codes using an inductive approach, allowing themes to emerge from the data. A coding framework will be used to categorize codes and identify meaningful and potential themes. We will perform peer debriefing regularly to discuss the emerging themes and interpretations, ensuring the rigor of the analysis.

### Quantitative Analysis

The epidemiological survey will be conducted on tablets with ODK software. All interviews will be uploaded electronically to Google Spreadsheets (Google Inc). The final data set will be downloaded as Excel spreadsheets. STATA/SE (version 16.1; Stata Corp) will be used for statistical analyses. A descriptive analysis will be conducted to summarize the important results. The sociocultural and demographic characteristics of the participants will be summarized. Illness-related questions, such as perceived causes, distress patterns, and help-seeking behaviors, will be presented and compared between different gender and age groups across study areas. The explanatory variables in this study will be social and cultural features of illness experience, meaning and behavior, and questions of awareness and use of the COVID-19 vaccines for prevention. Inferential analysis using regression models will assess the determinants of COVID-19 vaccine acceptance. Narratives of statistically significant factors will be reviewed to better understand their importance.

### Ethical Approval

The study has been approved by the Medical Research Advisory Committee of PNG (MRAC#22.19), the Medical Research Committee of Madang PHA (MAPHAREC 06.22), and the Research and Ethics Committee of Eastern Highlands Province PHA. All participants will provide written informed consent in either English or Tok Pidgin before participating in the study. In the case of illiterate participants, verbal informed consent will be obtained in the presence of a family member or relative. The purpose, procedure, and potential risks of the study will be explained to the participants, and they will be informed that they can withdraw from the study at any time without consequences.

## Results

The project proposal has been reviewed and approved by the Medical Research Advisory Committee of PNG. The permissions required from the provincial health authority to conduct data collection in the selected provinces have been obtained. The research team has recruited participants for in-depth interviews, including key informants in the communities and health facilities. The qualitative data collection started in December 2022, and the preliminary analysis of these data is underway. The insights from the in-depth interviews will be used to further refine the survey instrument by adapting it to the local context. We plan to pretest the survey instrument for validity and proceed with the survey in May 2023. The findings from the interviews and the survey will be disseminated to the participating communities and key stakeholders in a professional dissemination meeting, followed by publication.

## Discussion

### Expected Outcomes

This study’s experiences and findings will contribute to the effective planning and implementation of the COVID-19 vaccine program in PNG. Furthermore, our findings and approach to health social science vaccine research will have implications and potential applications elsewhere for strategic planning of effective action for other vaccines. The following are anticipated specific contributions of the study: (1) identify priorities and approaches for community action based on Melanesian culture to contribute to health system preparedness for control of pandemics; (2) understand the awareness and acceptance of vaccines for COVID-19, indicating the potential value of COVID-19 vaccines in PNG; (3) clarify current views of clinicians and other health professionals concerning their enthusiasm or reluctance to promote vaccine action to control COVID-19; (4) develop and clarify the value of an approach to studying vaccine acceptance in local settings so that planning may consider the relevance of cultural features of health and illness on anticipated and actual vaccine acceptance; (5) clarify features and determinants of COVID-19 vaccine acceptance that can guide policy to ensure that efficacious vaccines are also effective in the context of actual public health action (findings should guide strategic planning to address knowledge gaps and misunderstandings about the benefits and risks of COVID-19 vaccines); and (6) contribute to the National Research Agenda of PNG to strengthen the operation and delivery of the health system and services.

### Potential Impact on Participants

It is possible that participating in the study may influence participants’ views on COVID-19 vaccines. For example, exposure to information about the COVID-19 vaccine or discussions about vaccine hesitancy during data collection may lead participants to change their attitudes or beliefs about the vaccine. To mitigate any unintended consequences and ensure the ethical conduct of the study, we will maintain (1) informed consent, (2) accuracy of information, (3) voluntary participation, and (4) confidentiality. First, all participants will be provided with detailed information about the study and its purpose and will be asked to provide informed consent before participating. This will ensure that participants fully understand the nature of the study and the potential impact it may have on their views. Second, all participants will be provided with accurate and unbiased information about the COVID-19 vaccine, both before and after participating in the study. This will help to ensure that participants have access to the information they need to make informed decisions about the vaccine. Third, participation in the study will be completely voluntary, and participants will be free to withdraw from the study at any time without penalty. This will help to minimize any potential for coercion or undue influence on participants. Fourth, all data collected during the study will be kept confidential and participants’ identities will be protected. This will help to ensure that participants feel comfortable sharing their views and that their privacy is respected.

By taking these steps, we hope to minimize any unintended consequences of the study design on participants’ views on COVID-19 vaccine acceptance and ensure that the study is conducted ethically and with the informed consent of participants.

### Limitations

The proposed study design does not include any provinces from the Southern or Islands regions of PNG. We recognize that the generalizability of our study findings will be limited to the Highlands and Coastal regions. However, the Southern and Islands regions can also be categorized as coastal areas. In addition, this study might be subject to social desirability bias. In general, participants will provide responses that they believe are socially acceptable, rather than responding truthfully. To address this potential bias, open-ended questions and follow-up probes will be used to encourage participants to provide detailed and honest responses. However, some participants may still be hesitant to disclose certain information. In addition, the study will be limited by the potential impact of stigma on recruitment and participation. Participants might be hesitant to participate or disclose certain information due to concerns about stigma or judgment. Efforts will be made to address these concerns, including providing a safe and confidential environment for participation, but the impact of stigma on the findings will be considered when interpreting the results.

## Abbreviations

CEPI: Coalition for Epidemic Preparedness Innovations
COVAX: COVID-19 Global Vaccine Access
GAVI: Global Alliance for Vaccine and Immunization
LMICs: low- and middle-income countries
NCC: National Control Centre
NPI: nonpharmaceutical interventions
ODK: Open Data Kit
PHA: provincial health authority
PNG: Papua New Guinea
SAGE: Strategic Advisory Group of Experts
UNICEF: United Nations Children’s Fund
WHO: World Health Organization

## Data Availability

The data sets generated and/or analyzed during this study will be available in subsequent papers.
